# Efficacy and Safety of LetibotulinumtoxinA in the Treatment of Moderate and Severe Glabellar Lines in Females 35 to 50 Years of Age: Post Hoc Analyses of the Phase 3 Clinical Study Data

**DOI:** 10.1093/asjof/ojae010

**Published:** 2024-02-23

**Authors:** Michael Gold, Susan Taylor, Daniel S Mueller, Jeffrey Adelglass, Joely Kaufman-Janette, Sue E Cox, Michael Cecerle, Konstantin Frank, Mark Nestor

## Abstract

**Background:**

Botulinum toxin type A (BoNT-A) injections continue to be widely used as a common treatment for both males and females. According to a recent survey conducted by the International Society of Plastic Aesthetic Surgeons, the majority of patients receiving these injections are females between the ages 35 and 50.

**Objectives:**

A post hoc analysis was conducted to examine whether there were variances in the effectiveness and safety of letibotulinumtoxinA for treating vertical glabellar lines between the broader female study population and a particularly defined group of female participants aged 35 to 50.

**Methods:**

For this post hoc analysis, data from females aged 35 to 50 were extracted and analyzed from the BLESS III study. In this Phase 3 clinical trial, 355 participants with moderate-to-severe glabella frown lines received either 20 U of letibotulinumtoxinA or a placebo. The study evaluated Glabella Line Severity (GLS) score, treatment onset, duration of effects, time to retreatment, and adverse events. A positive response was determined by achieving a GLS score of 0 or 1, as assessed by both patients and investigators, along with at least a 2-point improvement in GLS score relative to baseline at Week 4 after the injections.

**Results:**

Composite responder rates for patients aged 35 to 50 receiving active treatment were significantly higher than for the remaining female population receiving active treatment at Weeks 1, 2, and 4. Females aged 35 to 50 showed higher rates of GLS improvement of ≥1 at Weeks 1, 2, 4, 8, 12, 16, and 20 compared with the remaining female population receiving active treatment. At Week 4, a higher percentage of females aged 35 to 50 achieved a GLS score of 0 upon maximum frowning compared with the remaining females. Females aged 35 to 50 had a shorter median time to onset of GLS improvement compared with the remaining female population. Safety assessments showed a low incidence of treatment-related adverse events in females aged 35 to 50.

**Conclusions:**

LetibotulinumtoxinA showed significantly higher response rates in females aged 35 to 50 compared with other female patients at Weeks 1, 2, and 4. Response rates remained higher up to Week 16. The treatment demonstrated efficacy and safety in treating vertical glabellar lines in this patient group.

**Level of Evidence: 2:**

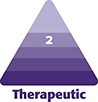

Injections with botulinum toxin type A (BoNT-A) remain one of the most common treatments for both males and females. A recent survey by the International Society of Plastic Aesthetic Surgeons (ISAPS) has revealed that the most treated patients are females 35 to 50 years of age. In 2021, of the 6.2 million performed injections with BoNT-A, a staggering 3.1 million procedures were performed in 35- to 50-year-old, mostly female, patients.^1^ The increasing demand of 26.0% for BoNT-A treatments between 2016 and 2020 can be attributed to the increasing acceptance of aesthetic interventions, rising self-awareness and, of course, the increasing availability of aesthetic treatments.^2,3^ Moreover, the presence of glabellar lines, forehead lines, and crow's feet lines is among the most concerning age-related changes of facial appearance and is all aesthetic conditions that are treated in most of the cases with BoNT-A were reported in a recent investigation.^4^ Along with the constantly rising demand for neuromodulator treatments,^5-9^ the number of available neuromodulators likewise has increased. A novel BoNT-A is letibotulinumtoxinA (Hugel, Inc., Chuncheon, Republic of Korea and CROMA Pharma, Leobendorf, Austria), a 900 kDa BoNT-A complex derived from the *Clostridium botulinum* strain CBFC26. Recently published data from a Phase 3 clinical trial of letibotulinumtoxinA has shown remarkable efficacy and safety, revealing comparable performance with other approved BoNT-As.^10^ However, the published analysis included the entire enrolled study population of male and female patients between ages 18 and 75. This included patients significantly older than in most other Phase 3 reports, which looked at patients aged up to 65 years, when reporting the efficacy and safety data. Specific subgroups, especially focusing on differences between millennials and non-millennials, on differently stratified age—groups were analyzed in recent publications.^11,12^ Although this represents a more narrow range of patients, accounting for individual differences in the response to neuromodulator treatments, according to the aforementioned results of the ISAPS survey,^1^ the most commonly treated patient group is female and aged 35 to 50. Thus, this post hoc analysis was performed to assess potential differences in the efficacy and safety of letibotulinumtoxinA in the treatment of vertical glabellar lines between the overall female study population and this very specific patient group of females aged 35 to 50, focusing on the most commonly treated patient group.

## METHODS

### Trial Design of BLESS III

The details of the completed trial, including design, institutions, and ethical setup, have been described in a previous publication.^10^ Briefly, 355 patients were enrolled in the prospective, randomized, parallel group, double-blind, multicenter, placebo-controlled Phase 3, which consisted of a double-blind phase and an open-label extension, lasting in total up to 60 weeks between May 2019 and December 2020. Ethical approval was granted by a central IRB (Copernicus, Cary, NC; IRB Number 20190353). In addition, the US FDA, the Austrian independent ethics committee (Medizinische Universität Wien), and a competent authority also approved the protocol prior to its initiation. Eligible patients were randomized at baseline (3:1 randomization scheme) to the active treatment or placebo group in the double-blind phase. The active product and the empty placebo vials were reconstituted using 1.25 mL of sterile physiological saline. Patients assigned to the active treatment group received a total of 20 U letibotulinumtoxinA, with a total injection volume of 0.5 mL, spread equally over 5 predefined intramuscular injection sites with an injection volume of 0.1 mL each, equivalent to 4 U per injection site. The injection sites included 2 injections in each corrugator supercilii muscle and 1 injection in the procerus muscle. The injection points and injection volume stayed the same in the patients assigned to the placebo group; however, saline was injected only. The enrolled patients received a maximum of 4 treatment cycles, consisting of a single treatment in the double-blind phase, and up to 3 subsequent treatments in the open-label extension phase. The double-blind phase lasted at least 12 weeks and was terminated upon qualification for retreatment of the patient. All patients were allowed to proceed into the open-label extension phase. In this phase, all patients were treated with letibotulinumtoxinA (20 U) using the same injection pattern and injection volume. Evaluation for eligibility for retreatment was performed at the earliest at 12 weeks after the first/previous treatment, according to retreatment criteria. Patients who did not qualify for retreatment at Week 12 were offered retreatment once eligible at a later visit (at 4 weekly intervals thereafter) up until 48 weeks. In the double-blind phase, patients attended follow-up visits at 1, 2, and 4 weeks, and at 4 weekly intervals thereafter for the evaluation of efficacy and safety. After retreatment, patients attended follow-up visits after 1 and 4 weeks and at 4 weekly intervals thereafter. At Week 2, an additional telephone visit was conducted, and the Week 8 visit was also performed as telephone visit. Efficacy measurements included assessment of glabellar line severity using a 4-point glabellar line severity scale (0 = none, 1 = mild, 2 = moderate, 3 = severe). Safety included investigator assessment of adverse events. For further information in regards of measurements performed during the trial, please refer to the original publication.^10^ The subgroup analysis was conducted based on the initial study. The Clinicaltrials.gov registration number is NCT03985982.

### Statistical Post Hoc Analyses

All statistical procedures were performed using Statistical Analysis Software version 9.4. For this post hoc analysis, data from females aged 35 to 50 were extracted and analyzed from the BLESS III study. Comparisons with the remaining female patient pool of the BLESS III investigation (age <35 or >50 years) were performed using 2-sided *P*-values based on exact unconditional tests. Two-sided 95% CIs were provided when relevant. Continuous variables were summarized using descriptive statistics, including number of patients (*n*), mean, standard deviation, median, minimum, and maximum. For categorical variables, summaries included counts of patients and percentages in corresponding categories. The full analysis set population was used for the evaluation of the efficacy assessments. The safety analysis set was used for the evaluation of the safety assessments.

## RESULTS

### Patient Disposition

Of the 355 patients enrolled, a total of 328 females fulfilled the requirements for this post hoc analysis. One hundred and sixteen females were aged between 35 and 50 years, whereas 212 females were younger than 35 years or older than 50 years. Females aged between 35 and 50 years had a mean age of 43.6 ± 4.7 years (range, 35-50 years) and a mean BMI of 25.26 ± 4.6 kg/m^2^ (range, 35-50 kg/m^2^), whereas the remaining females had a mean age of 55.4 ± 11.8 years (range, 21-75 years) and a mean BMI of 27.05 ± 5.4 kg/m^2^ (range, 15.1-55.8 kg/m^2^). Of the females aged 35 to 50 years, 87 patients were randomized to the active treatment group and 29 patients were randomized to the placebo group, whereas of the remaining female patients, 161 were randomized to the active treatment group and 51 to the placebo group. Fitzpatrick Skin Types I, II, III, IV, V, or VI were reported for 2 (1.7%), 39 (33.6%), 40 (43.5%), 27 (23.3%), 7 (6.0%), and 1 (0.9%) 35- to 50-year-old females, respectively. In the double-blind phase active treatment group, 24 (27.6%) 35- to 50-year-old females presented with moderate glabellar lines, whereas 63 (72.4%) 35- to 50-year-old females presented with severe glabellar lines, as assessed by the investigator. Of the remaining females, 39 (24.2%) presented with moderate and 122 (75.8%) with severe glabellar lines. Further demographic variables are summarized in Table. In the 35- to 50-year-old age group, the primary reason for discontinuation from the study during the double-blind phase was withdrawal by patient (3 patients), whereas in total, 7 patients did not complete the double-blind phase. Of the females younger than 35 or older than 50 years old, 13 did not complete the double-blind phase, with withdrawal by patient being the most common reason for discontinuation (7 patients).

**Table.** Demographic Information of the Female Patients Analyzed Aged 35 to 50 Years and Younger Than 35 or Older Than 50 Years

	35 to 50 years old	<35 and >50 years old
*n*	116	212
*Age*		
Mean	43.6	55.4
SD	4.7	11.8
Minimum	35	21
Median	44	57
Maximum	50	75
*BMI*		
Mean	25.26	27.05
SD	4.6	5.4
Minimum	16.2	15.1
Median	24.15	26.05
Maximum	39.9	55.8
*Fitzpatrick skin type*		
I	2 (1.7)	7 (3.3)
II	39 (33.6)	87 (41.0)
III	40 (34.5)	62 (29.2)
IV	27 (23.3)	34 (16.0)
V	7 (6.0)	16 (7.5)
VI	1 (0.9)	6 (2.8)
*Baseline GLS at maximum frown as assessed by investigator*		
Moderate	32 (27.6)	51 (24.1)
Severe	84 (72.4)	161 (75.9)

SD, standard deviation; GLS, glabella line severity.

### Efficacy

**Figure 1.**
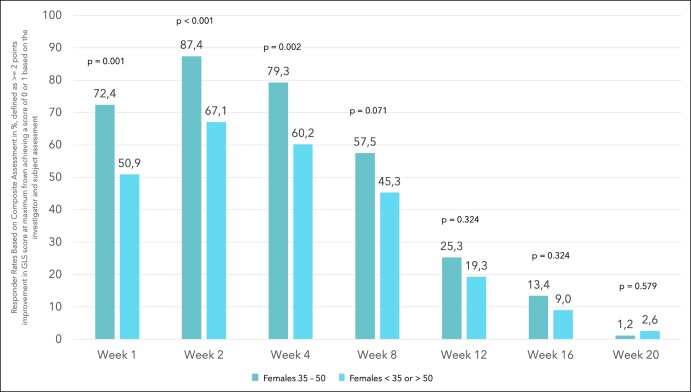
Bar diagram showing the response rate in % for females aged 35 to 50 years and younger than 35 or older than 50 years based on the composite assessment.

**Figure 2.**
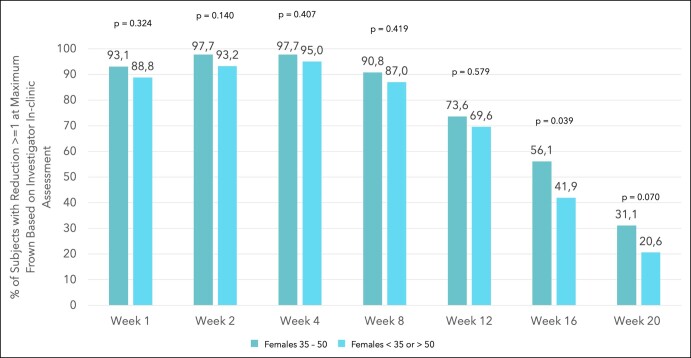
Bar diagram showing the % of patients with reduction ≥1 at maximum frown based on investigator in-clinic assessment for females aged 35 to 50 years and younger than 35 or older than 50 years.

Composite responder rates, where response was defined as ≥2-point improvement in Facial Wrinkle Scale score at maximum frown achieving a score of 0 or 1 based on the investigator and patient assessment, of the patients enrolled in the active treatment group were 72.4%, 87.4%, 79.3%, 57.5%, 25.3%, 13.4%, and 1.2% at Weeks 1, 2, 4, 8, 12, 16, and 20 compared with 50.9%, 67.1%, 60.2%, 45.3%, 19.3%, 9.0%, and 2.6% in the remaining female study population (Figure 1). Statistically significant differences in the composite responder rates between females aged 35 to 50 years and the remaining female study population were found at Weeks 1, 2, and 4 with *P* < .05. The differences in responder rates between females aged 35 to 50 years and the remaining female study population were 21.48 (95% CI, 7.33-33.27), 20.28 (95% CI, 8.20-30.09), 19.06 (95% CI, 5.60-30.07), 12.13 (95% CI, −1.23 to 24.99), 6.03 (95% CI, −4.72 to 17.78), 4.38 (95% CI, −3.89 to 14.42), and −1.38 (95% CI, −5.59 to 4.28) at Weeks 1, 2, 4, 8, 12, 16, and 20. Based on the investigator's assessment 93.1%, 97.7%, 97.7%, 90.8%, 73.6%, 56.1%, and 31.3% of the females aged 35 to 50 years in the active treatment group presented with a Glabella Line Severity (GLS) improvement of ≥1 at Weeks 1, 2, 4, 8, 12, 16, and 20, compared with 88.8%, 93.2%, 95.0%, 87.0%, 69.6%, 41.9%, and 20.6% in the remaining female population in the active treatment group (Figure 2). A statistically significant difference between the 2 groups was determined at Week 16 with *P* = .039. Differences of responder rates between females aged 35 to 50 years and the remaining female study population were 4.28 (95% CI, −4.25 to 11.49), 4.53 (95% CI, −2.07 to 10.10), 2.67 (95% CI, −3.75 to 7.79), 3.85 (95% CI, −5.40 to 11.70), 4.00 (95% CI, −8.35 to 15.47), 14.16 (95% CI, −0.31 to 27.28), and 10.68 (95% CI, −1.24 to 23.10) at Weeks 1, 2, 4, 8, 12, 16, and 20.

At Week 4, based on the investigator assessment, 73.6% of the females aged 35 to 50 years had a GLS score of 0 (none) upon maximum frowning, whereas 52.2% of the remaining females presented with a GLS score of 0 (*P* = .001).

Median time to onset of ≥1-point improvement in GLS from baseline was 2.8 days in the females aged 35 to 50 years, whereas it was 4.2 days in the remaining female population with *P* < .001, as assessed by the diary data of the patients. More than half of the 35- to 50-year-old female patients were responders up to Week 16 (56.1%), whereas in the remaining female population, more than half of the patients were responders up to Week 12 (69.6%).

### Safety Assessment

No treatment emergent adverse events (TEAE) led to study discontinuation in females aged 35 to 50 years. In the double-blind phase, a total of 20 patients reported any TEAE, of which, 1 was a severe TEAE, 1 study drug, and 1 other injection related (1.1%, each). The study-drug-related and also the injection-related TEAE were reported to be headache.

## DISCUSSION

This subanalyses of females focused on the efficacy and safety of an injection of 20 U letibotulinumtoxinA into the glabella complex in females aged 35 to 50 years and compared the results to the remaining female patient pool of the BLESS III study.

**Figure 3.**
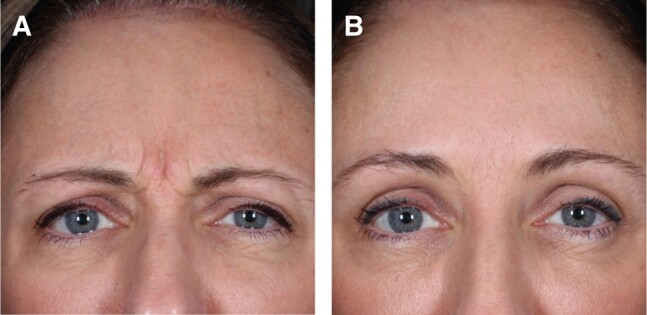
A 38-year-old female patient from the 35- to 50-year-old age group with severe glabellar lines at baseline (A) and no glabellar lines after 4 weeks (B).

**Figure 4.**
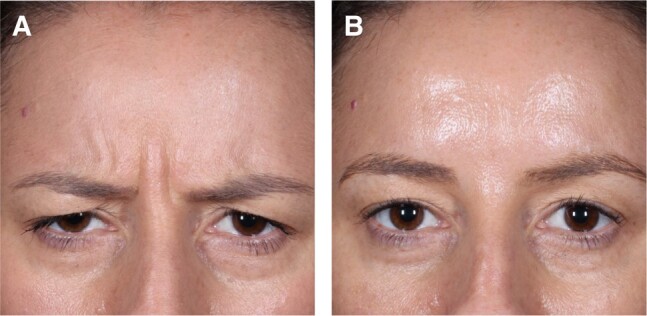
A 46-year-old female patient from the 35- to 50-year-old age group with severe glabellar lines at baseline (A) and no glabellar lines after 4 weeks (B).

The results revealed a significantly higher response rate based on the composite endpoint of a ≥2-point improvement in facial wrinkle score at maximum frown achieving a GLS score of 0 or 1 (investigator and patient in-clinic assessment) at Weeks 1, 2, and 4 for females aged 35 to 50 years compared with the remaining female patients (Figures 3, 4). Moreover, response rate was higher up to Week 16 for females aged 35 to 50 years, when comparing the composite endpoint, reflected by the difference in responder rates and the 95% CIs. A greater proportion of patients with a ≥1-point improvement in GLS at maximum frown based on the investigator assessment was observed in females aged 35 to 50 years, compared with the remaining female population up to Week 20. At Week 16, the difference in response rate between females aged 35 to 50 years and the remaining female population reached statistical significance. Although a clinically meaningful amelioration of vertical glabellar line severity, defined by a ≥1-point improvement, has been shown in a majority of all female patients up to Week 12, more than half of the females aged 35 to 50 presented with a ≥1-point improvement up to Week 16. Especially the Week 4 findings, reporting a response rate of 97.7% in 35- to 50-year-old females underlines the remarkable efficacy of the active drug for the treatment of vertical glabellar lines. Although letibotulinumtoxinA has already shown remarkable performance when investigating the entire study population,^10^ it needs to be pointed out that the female patients aged 35 to 50 years showed exceptional response between Week 2 and Week 8.

A median onset of ≥1-point improvement in GLS from baseline was 2.8 days in the active treatment group, based on the patient's diary data. Onset as defined was 4.2 days in the remaining female population, which is in line with prior studies investigating the onset of incobotulinumtoxinA, abobotulinumtoxinA, and onabotulinumtoxinA in general populations, including females and males. Kane et al reported a median onset of 3.5 days after treatment with incobotulinumtoxinA and 3.9 days after treatment with onabotulinumtoxinA.^13^ Carruthers et al observed a median time of onset of a clinically meaningful response at 3 days after treatment with daxibotulinumtoxinA.^14^ Kane et al reported a median time of onset of 4 days after injection with abobotulinumtoxinA,^15^ whereas Carruthers et al observed a median onset at 2 days after injection with incobotulinumtoxinA.^16^

The duration of letibotulinumtoxinA, defined as time point, where a majority of patients still responded to the treatment (≥1-point improvement compared with baseline) was 16 weeks in females aged 35 to 50 years and 12 weeks in the remaining female population, which reflects the clinically common interval between treatment and re-treatment.

No randomized, placebo-controlled investigation compared the efficacy and safety of BoNT-A injections in females aged 35 to 50 years to females younger than 35 and older than 50. The relevance of focusing on this specific patient group is the high number of treatments performed in this patient group, as pointed out by a recent investigation of the ISAPS. Although the definition of millennials is not distinctly defined, most commonly the definition of a millennial encompass patients who were born between 1981 and 1996, that is, patients aged between 27 and 42 years, which only partially intersects with the most commonly treated group of patients. It is thus of importance not to rely on sole generational definitions, but rather to assess a distinctly defined age group, as millennials will become patients aged 35 to 50 in 8 years from now on, which will ultimately alter their response to utilized neuromodulations, due to physiological change in the facial musculature. Although most Phase 3 studies include a wide range of patients in terms of age, it needs to be noted that the mandatory dosage might not always reflect the actual clinical demand of the patient. Evidently, many neurophysiological changes occur with increasing age, which will affect the efficacy of neuromodulators. Although not completely identified, focal denervation of the neuromuscular junction seems to be the driving process of the decreasing number of acetylcholinergic receptors, as well as decrease in size and number of acetylcholinergic end plates.^17-19^ Changes in the facial muscle could also been demonstrated in recently published in vivo electromyography investigations.^7^ This could potentially explain the slightly better outcome in females aged 35 to 50 years, as elderly patients might require a higher dosage due to the aforementioned age-dependent processes at the neuromuscular junction. This subanalysis has shown that in the most frequently targeted patient group, facing a certain degree of aging that can be considered mid-aged, 20 U of letibotulinumtoxinA, spread over 5 injection points offers high efficacy with minimal complication rates.

Physicians can rely on the even (significantly) higher efficacy of letibotulinumtoxinA in the treatment of glabellar lines in females aged 35 to 50, whereas it is also well tolerated, as shown by the remarkable safety findings. No eyelid ptosis was reported in this investigation after injection of letibotulinumtoxinA, which is noteworthy, as recent investigations have reported occurrence rates of eyelid ptosis with a frequency ranging between 0.51% and 5.4% for other toxins.^20^ It remains to be investigated, whether letibotulinumtoxinA did not cause any eyelid ptosis due to a potentially more focused and precise diffusion pattern. The higher response rates in females aged 35 to 50 suggest that LetibotulinumtoxinA could be a preferred choice for treating glabellar lines in this age group. This age-specific efficacy, coupled with the safety profile observed, provides a valuable guide for practitioners in tailoring treatments to achieve optimal outcomes.

This subanalysis is not free of limitations. Although considered as the gold standard, the assessments of efficacy using validated scales still remain subjective to a certain extent and do not completely objectify the outcome of a treatment. Three-dimensional surface imaging and vectorial skin displacement might reveal more precise data; however, as none of these measurement techniques have been validated or accepted by regulatory organs, assessment using validated scales was chosen. Moreover, the percentage of patients with skin of color was underrepresented when compared with those without skin of color. The decision to subdivide the female cohorts into 2 groups—those aged 35 to 50 and everyone else—may potentially skew comparisons, particularly given the inherent differences in wrinkle prevalence and severity across different age groups. This aspect should be considered when interpreting our findings and underscores the need for further research with more granular age stratification. In addressing the limitations of our study, we acknowledge that multiple χ^2^-like tests were performed without applying a post hoc correction for multiple testing. Although this decision aligns with our study's exploratory nature and the specific objectives set forth, it does raise the potential for Type I errors (false positives). Our approach was to prioritize sensitivity in hypothesis generation over the strict control of Type I errors. However, this decision means that some of the observed statistical significance might be due to chance. This aspect should be considered when interpreting our findings, particularly in the context of formulating clinical guidelines or designing further confirmatory studies.

## CONCLUSION

The results revealed a significantly higher response rate based on the composite endpoint of a ≥2-point improvement in facial wrinkle score at maximum frown achieving a GLS score of 0 or 1 (investigator and patient in-clinic assessment) at Weeks 1, 2, and 4 for females aged 35 to 50 years compared with the remaining female patients. Moreover, response rate was higher up to Week 16 for females aged 35 to 50 years old, when comparing the composite endpoint, reflected by the difference in responder rates and the 95% CIs. Overall, it can be concluded that letibotulinumtoxinA showed remarkable efficacy and safety in the treatment of vertical glabellar lines in the most commonly targeted patient group.
